# Challenges in zero‐flow blood pressure as a measure of mean circulatory filling pressure: Experimental and computational perspectives

**DOI:** 10.14814/phy2.70599

**Published:** 2025-10-03

**Authors:** L. M. van Loon, M. P. Mulder, D. van Dort, J. G. van der Hoeven, D. W. Donker, J. Lemson

**Affiliations:** ^1^ Cardiovascular and Respiratory Physiology Technical Medical Centre, University of Twente Enschede The Netherlands; ^2^ Department of Intensive Care Medicine Radboud University Medical Center Nijmegen The Netherlands; ^3^ Intensive Care Center University Medical Center Utrecht Utrecht The Netherlands; ^4^ Department of Cardiology Radboud University Medical Center Nijmegen The Netherlands; ^5^ Radboud Center for Infectious Diseases Nijmegen The Netherlands

**Keywords:** baroreflex, computational physiological modeling, fluid therapy, hemodynamics, venous return

## Abstract

Mean circulatory filling pressure (MCFP) is a foundational hemodynamic concept, representing the average circulatory pressure in a no‐flow state and used to assess volume status and venous return. However, its measurement and clinical relevance remain debated. In this study, zero‐flow pressures were recorded in 14 healthy pigs following cardiac arrest. Measurements were taken for 10 min in the abdominal aorta and right atrium under two conditions: cardiac arrest induced by pentobarbital overdose or by ventricular fibrillation (VF), with half of the VF animals rendered hypovolemic. A validated computational model of cardiovascular physiology was used to simulate these scenarios. Pentobarbital caused a rapid pressure equilibration, with mean arterial pressure (MAP) falling from 47 ± 3.7 to 16 ± 2.5 mm Hg. In contrast, VF produced a dynamic pressure response: MAP dropped from 53 ± 4.7 to 17 ± 2.2 mm Hg while central venous pressure rose, producing a persistent retrograde pressure gradient (5 ± 2.1 mm Hg). In silico simulations closely matched these dynamics (normalized RMSE <5%) and confirmed the influence of reflex mechanisms. These results challenge the idea of MCFP as a stable, universal value. Zero‐flow pressures depend heavily on arrest method and reflexes, and computational modeling offers a valuable, ethical alternative to animal‐based investigations.

## 1 INTRODUCTION

The mean circulatory filling pressure (MCFP) is considered a critical hemodynamic parameter in its role as the upstream pressure in Guyton's model of venous return (Guyton et al., 1955). This pressure is theoretically defined as “the pressure that would be measured at all points in the entire circulatory system if the heart were stopped suddenly and the blood were redistributed instantaneously in such a manner that all pressures were equal.” (Guyton et al., 1973). However, accurately capturing this pressure in practice is challenging, as the measurement must occur within seconds after cardiac arrest to reflect the true MCFP (Guyton et al., 1954). Moreover, the very premise of teaching or demonstrating MCFP is inherently troublesome, as it requires cardiac arrest—an intervention that is both ethically and practically untenable in clinical settings.

Although zero‐flow pressures will therefore never have direct bedside applicability, they remain important in two ways. First, historically, they provided the conceptual foundation for Guyton's model of venous return. Second, they continue to serve as a research tool: in experimental settings, MCFP and its surrogates have been used to study stressed volume, vascular compliance, and fluid responsiveness, and to provide a “ground truth” against which new hemodynamic methods can be validated (Cooke et al., 2018; Gaddis et al., 1986; Maas 2012).

The dependence of intravascular pressure on external factors such as wall stress, blood volume, and vasomotor activity has led to its interpretation as a surrogate marker for intravascular volume status and fluid responsiveness in the critically ill. While the concept of MCFP is foundational in a great number of hemodynamic studies, practical measurements have often been confounded by methodological issues, particularly those that inadvertently alter blood volume or vasomotor tone during the induction of cardiac arrest. These challenges cast doubt on the validity of MCFP as a stable and uniform pressure and raise questions about its clinical relevance, especially in dynamic and critical physiological conditions.

In this study, we adopted an integrative approach combining experimental animal models with in silico cardiovascular simulations. The experimental part involved systematic assessment of zero‐flow blood pressures under various conditions, including different volume conditions and modes of cardiac arrest induction. The computational model was specifically designed to simulate the hemodynamic responses observed experimentally, providing a controlled environment to test hypotheses that are difficult or impossible to explore in vivo.

This dual approach allows us not only to validate experimental findings through computational simulations but also to dissect the underlying mechanisms that contribute to the observed phenomena. By integrating these methodologies, we aim to enhance our understanding of the MCFP, particularly how the method of cardiac arrest induction influences the recorded blood pressures. The purpose of this study is to test the hypothesis that the mechanism used to experimentally arrest the heart significantly influences the estimated MCFP.

## 2 MATERIALS AND METHODS

This experiment was performed after approval of the local ethics committee on animal research at the Radboud University Nijmegen Medical Center (RUNMC License number RU‐DEC 2014‐246) and in full compliance with Dutch and European legal requirements on the use and protection of laboratory animals. In the context of the principles of replacement, reduction and refinement for the use of animal models, animals used in the study were previously used for medical training, placing a neural flow diverter (not affecting the cardiovascular system nor neuro‐vascular responses).

The reported results are a follow‐up of a previous experiment in which we studied the applicability and validity of a bedside technique to estimate mean systemic filling pressure. A total of 14 piglets (female, aged 3–6 months, mean weight of 41.5 kg [range: 31–55 kg], mean body surface area of 3.1 m^2^ [range: 2.2–3.8 m^2^]) under general anesthesia were studied. For a detailed description of the anesthesia, ventilation, and surgical preparation, we refer to the materials and methods section of our previous publication (van Loon et al., 2020).

### 2.1 Induction

In summary, premedication consisted of the intramuscular administration of 10 mg/kg midazolam (Roche, Woerden, The Netherlands), 1 mg/kg ketamine (Eurovet Animal Health BV, Bladel, The Netherlands), 50 μg/kg atropine (Pharmachemie BV, Haarlem, The Netherlands) and 20 μg/kg amoxicillin (Centrafarm Services B.V., Etten‐Leur, The Netherlands). Anesthesia was induced using 2 mg/kg IV administration of propofol (B. Braun Melsungen AG, Melsungen, Germany) and maintained using inhalation of 0.5–2 volume% isoflurane (Baxter International, Deerfield, IL, USA), the continuous V administration of 10 (μg/kg)/h sufentanil (Janssen‐Cilag BV, Tilburg, The Netherlands) and 1 (mg/kg)/h rocuronium (*Organon*, Oberschleißheim, Germany) after a loading dose of 1 mg/kg. Lungs were mechanically ventilated using a volume‐controlled mode. All animals received a fluid bolus of 5 mL/kg at the end of instrumentation to prevent initial hypovolemia.

### 2.2 Measurements

Cardiac output (CO) was acquired using an ultrasound transit‐time perivascular flow probe (18 or 22 mm) (PAX series, Transonic Systems, Ithaca, NY) which was placed around the main pulmonary artery. Arterial blood pressure (ABP) was acquired using an indwelling thermistor‐tipped arterial catheter (4‐5Fr, PulsiocathTM, Pulsion Medical Systems SE) which was placed in the lower abdominal aorta via the right femoral artery. Central venous pressure (CVP) was acquired using a central venous catheter (5‐7F, 3‐lumen, 13 cm; Arrow International, Reading, PA) which was inserted via the right jugular vein for CVP recording. All signals were sampled at a rate of 200 Hz, A/D converted (NI USB‐6211, National Instrument, Austin, TX, USA), and stored on a hard disk. A fast‐flush test was performed prior to the experiment to verify the dynamic response of the pressure system.

Furthermore, the pressure transducers were leveled, zeroed, and placed on the same transducer bar.

### 2.3 Experimental protocol

**FIGURE 1:**
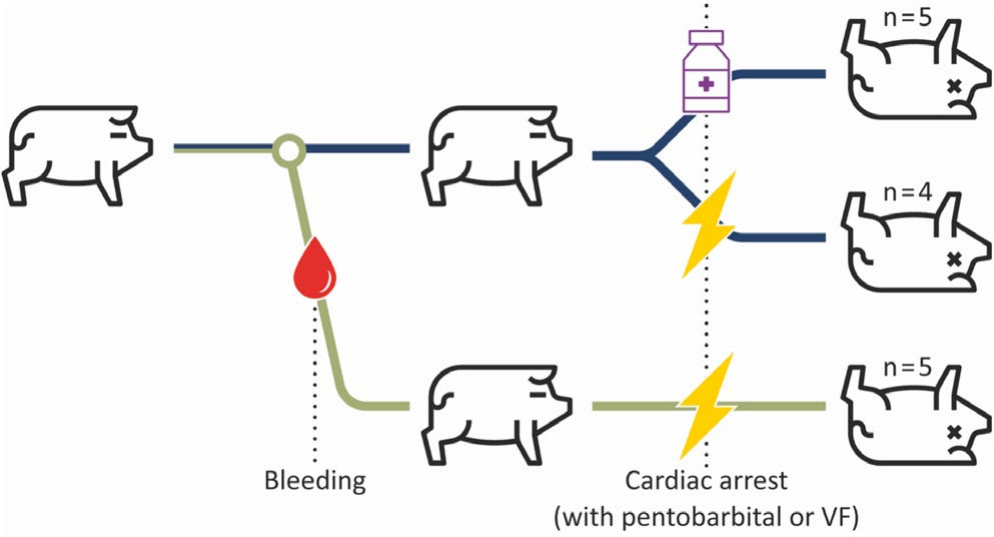
Schematic overview of experimental protocol. VF, ventricular fibrillation.

Cardiac arrest was induced either with an overdose of pentobarbital (150 mg/kg IV) or by ventricular fibrillation (VF) triggered with a 60‐Hz alternating current applied via cardiac pacing wires (Figure 1). Cardiac arrest was defined as the absence of a central pulse. Animals in the VF group were randomly assigned to euvolemic or hypovolemic conditions, with hypovolemia induced by withdrawal of 10 mL/kg blood volume. All animals in the pentobarbital group were euvolemic at the time of arrest, verified by a stable mean arterial pressure (>50 mmHg). Prior to arrest, the endotracheal tube was disconnected from the ventilator to achieve zero intrathoracic pressure. At the conclusion of the experiment, animals were euthanized with an intravenous overdose of pentobarbital (150 mg/kg) when not already administered for arrest induction.

### 2.4 Data recording and processing

After establishing cardiac arrest with no blood flow, CO, and CVP and ABP were continuously recorded for another 10 min. Mean ABP, CVP and CO were acquired by low‐pass filtering of the recorded signals (cut‐off frequency of 0.5 Hz (Steptoe et al., 1985), third order Butterworth filter applied in the forward and reverse direction for a zero‐phase response) using custom‐written MATLAB (MATLAB R2022a, The MathWorks Inc. Massachusetts, USA) scripts.

### 2.5 Computational model

A validated open‐source code computational physiological model was used, CVSim, which was developed for simulating human cardiovascular physiology (Heldt 2010) and is available through PhysioNet.org (Moody et al., 2000). In short, it is a lumped‐parameter computational model based on an electrical circuitry analogy consisting of 21 Windkessel units arranged as a closed‐loop system. Each of the 2‐element Windkessel units consists of a resistor and a capacitor, representing the resistance to the input and outflow and the capacitance of the flow compartment as a blood reservoir (volume). The blood pressure serves as the dependent variable and is simulated by the voltage at each node of the electrical circuit. The blood flow rate then can be calculated as the current passing through each resistor. The cardiac compartments alternate between minimum and maximum elastance driven by heart rate to generate pulsatile pressure. The influence of the respiratory system on the cardiovascular system was simulated by applying an intrathoracic pressure waveform to the intrathoracic compartments in the model.

The basic model is controlled by an arterial baroreflex (ABR) and cardiopulmonary reflex (CPR) as short‐term blood pressure regulation, representing the sympathetic and parasympathetic nervous system influences on the cardiovascular system. These reflexes were modeled in a stepwise manner to capture their dynamic effects on heart rate, contractility, peripheral resistance, and vascular compliance. The process was divided into four key steps: Step I: Pressure and pulse pressure from the aortic arch (compartment 0) and pressure from the right atrium (compartment 15) were integrated over 250 data points to smooth the signals. Step II: Error signals were created by subtracting predefined set points (95 mmHg for arterial pressure, 35 mmHg for pulse pressure, and 3 mmHg for venous pressure) from the integrated signals. These error signals were scaled using an inverse tangent function, following the method of deBoer et al., with scaling limits of 18 for ABR and 5 for CPR (deBoer et al., 1987). Step III: The scaled error signals were convolved with unit‐area impulse response functions to represent different reflex components of the autonomic nervous system. Step IV: The resulting vectors were multiplied by static gain values specific to each effector mechanism, influencing heart rate, contractility, peripheral resistance, and unstressed volume (Zong 2003).

### 2.6 Simulation scenarios

Initial model parameters describe a healthy adult male, including the cardiovascular reflex parameters, the resistances, elastances, and unstressed volumes as obtained from the literature (Heldt 2010). Four simulation scenarios were designed to reflect experimental conditions: euvolemia or hypovolemia (20% blood volume reduction, as in the animal experiments), combined with the arterial baroreflex (ABR) and cardiopulmonary reflex (CPR) inactive to mimic reflex activity in pentobarbital‐induced cardiac arrest. Zero‐flow conditions, as observed in both pentobarbital and VF‐induced cardiac arrest, were simulated by setting heart rate, respiratory rate, and cardiac elastance to zero (Mamorita et al., 2024).

### 2.7 Statistics

Statistical analysis was performed using MATLAB (MATLAB R2022a, The MathWorks Inc. Massachusetts, USA). Normality was assessed using the Shapiro–Wilk tests. Four discrete points of the continuous blood pressure curves were taken for statistical analysis for both the experimental and in silico data, representing physiologically relevant phases of the post‐arrest response (baseline, initial equilibration, peak, and plateau). At baseline (T0, approx. 20 s prior to the induction of cardiac arrest), when CVP and MAP equilibrated (T1, approx. 30 s after cardiac arrest), when CVP and MAP were at their maximum (T2, approx. 300 s after cardiac arrest), and 10 min after cessation of flow (T3, approx. 600 s after cardiac arrest). Parameter changes over time after cardiac arrest were analyzed using repeated measures one‐way ANOVA. Differences between the euvolemic VF‐group, hypovolemic VF‐group and Pentobarbital‐group over time, and between the two simulation settings were tested using repeated measures two‐way ANOVA (interaction term). Differences between venous and arterial blood pressure at 600 s after cardiac arrest were analyzed using paired Student's *t*‐tests. A two‐sided *p*‐value of <0.05 was considered statistically significant. To assess the comparability between experimental data and in silico simulations, normalized root mean square error (RMSE) was used, with values below 5% indicating a high degree of agreement between the two datasets.

## 3 RESULTS

### 3.1 Experimental results

All animals included in the study were considered healthy on physical examination when entering the animal laboratory and the cardiovascular system was unaffected. Baseline hemodynamic parameters (T0), 20 s prior to the induction of cardiac arrest, are summarized in Table 1.

**TABLE 1:** Experimental hemodynamic data before and after cardiac arrest.

	Baseline (*t* = −20 s)	Crossing (*t* = 30 s)	Peak (*t* = 300 s)	10‐min after VF (*t* = 600 s)	Over time^a^	Over time vs. Pento^b^	Vs. VF‐euvolemia^c^ (*t* = −20 s)	Vs. Pento^d^ (*t* = 600 s)
Pentobarbital‐euvolemia								
CO (L/min)	4.3 ± 1.2	0 ± 0	0 ± 0	0 ± 0	ns	na	ns	na
MAP (mm Hg)	47 ± 3.7	16 ± 2.5	14 ± 2.8	15 ± 2.9	ns	na	ns	na
CVP (mm Hg)	12 ± 2.9	15 ± 3.1	14 ± 3.3	14 ± 3.5	ns	na	ns	na
VF–euvolemia								
CO (L/min)	3 ± 1.2	0 ± 0	0 ± 0	0 ± 0	ns	ns	na	ns
MAP (mm Hg)	53 ± 4.7	17 ± 2.2	17.9 ± 1.6	11 ± 0.8	*p* < 0.01	*p* < 0.01	na	*p* < 0.01
CVP (mm Hg)	11 ± 6	15 ± 4.6	22.9 ± 5	16 ± 3.7	*p* < 0.01	*p* < 0.01	na	*p* < 0.01
VF–hypovolemia								
CO (L/min)	2.4 ± 0	0 ± 0	0 ± 0	0 ± 0	ns	ns	ns	ns
MAP (mm Hg)	45 ± 5.6	14.8 ± 1.1	16.2 ± 1.1	9 ± 1.6	*p* < 0.001	*p* < 0.001	*p* = 0.04	*p* < 0.001
CVP (mm Hg)	9 ± 3.3	13.3 ± 2.9	19.4 ± 3.1	13 ± 3.1	*p* < 0.001	*p* < 0.001	ns	*p* < 0.001

*Note*: Data are expressed as mean ± standard deviation. Experimental time points: *t* = −20s; baseline measurement, *t* = 30s; when CVP and MAP equilibrated, *t* = 300 s; when CVP and MAP were at their maximum value, *t* = 600 s; 10 min after cessation of blood flow.

Abbreviations: CO, cardiac output; crossing indicates; CVP, central venous pressure; MAP, mean arterial blood pressure; na, not applicable; ns, not significant; Pento, pentobarbital; VF, ventricular fibrillation.

^a^ Significant changes over time (*t* = 30 to *t* = 600 s) within group to test for dynamic behavior of blood pressure recording (repeated measures one‐way ANOVA).

^b^ Significant changes over time (*t* = 30 to *t* = 600 s) vs. corresponding parameter in the pentobarbital group (repeated measures two‐way ANOVA, time × treatment interaction term).

^c^ Paired Student's *t*‐tests versus corresponding parameter in the Pento group.

^d^ Paired Student's *t*‐tests versus corresponding parameter in the VF euvolemic group.

**FIGURE 2:**
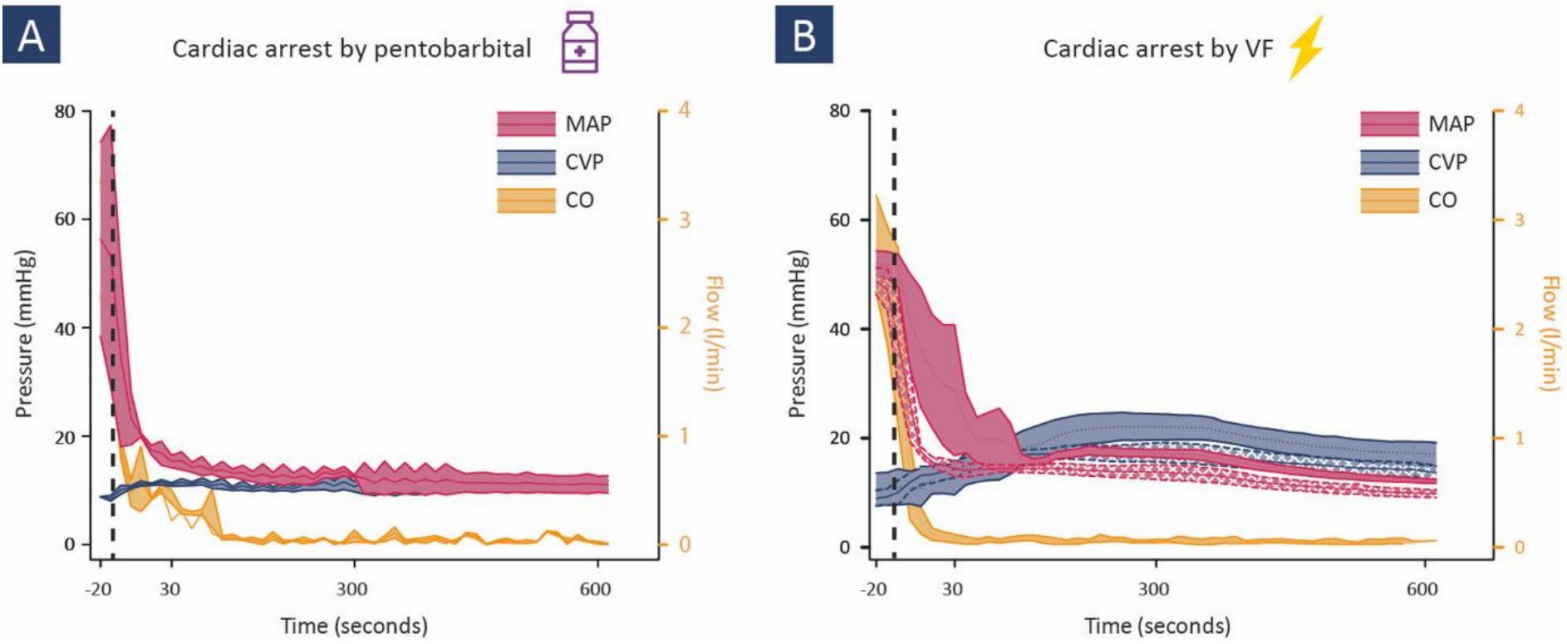
Experimental model revealing hemodynamic responses to zero‐flow. CO, cardiac output (CO), mean arterial (MAP) and central venous blood pressure (CVP) recording after cardiac arrest induced by pentobarbital (panel A) or ventricular fibrillation (VF, panel B). A vertical dashed line at time 0 marks the induction of cardiac arrest. Data are expressed as mean ± standard deviation.

The high pentobarbital dose in euvolemic piglets resulted in an instant drop in CO and MAP, and a rise in CVP. MAP and CVP came to‐and stayed in‐equilibrium 30 s after the onset of cardiac arrest (Figure 2b). The course of arterial blood pressure in this group was significantly different from that of that where cardiac arrest was induced by VF (Figure 2).

VF resulted in an instant drop in MAP and complete cessation of blood flow, while CVP increased (Figure 2a, Table 1). MAP became lower than CVP within 1 min after VF, which was not influenced by volume status. MAP and CVP started to increase until reaching a peak (± 300 s after VF initiation) before decreasing again. MAP and CVP reached a plateau within 10 min after VF, while a significant negative pressure difference (MAP‐CVP) persisted. Both MAP and CVP exhibited significant changes over time under both volume conditions, reflecting the dynamic hemodynamic behavior following VF‐induced cardiac arrest (Table 1, Figure 2b).

### 3.2 In silico simulations

**FIGURE 3:**
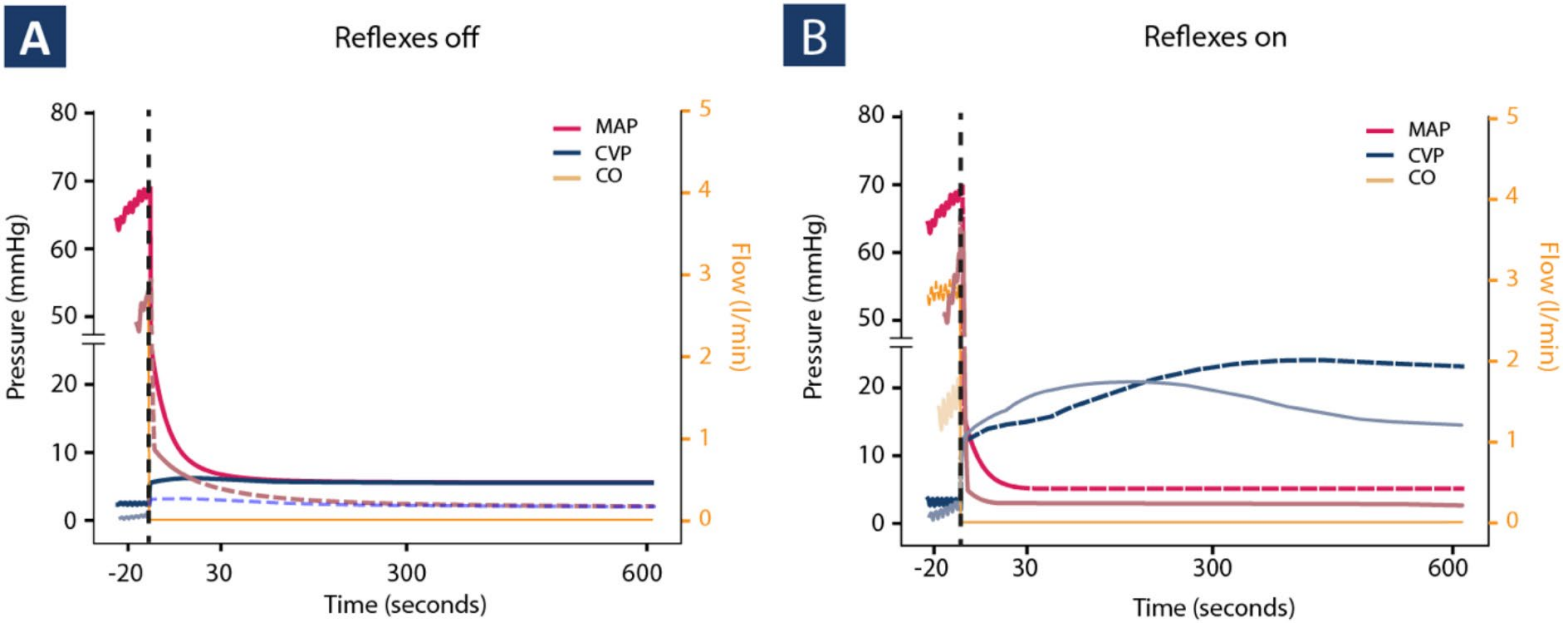
Computational model revealing hemodynamic responses to zero‐flow. Cardiac output (CO), mean arterial pressure (MAP) and central venous blood pressure (CVP) simulation results after setting heart rate to zero, with euvolemia (solid line) and hypovolemia (dashed line). (A) ABR and CPR reflex off, and (B) ABR and CPR reflex on.

Simulated mean blood pressure curves for both the arterial and venous side together with the cardiac output are shown in Figure 3. When the reflex mechanisms remained unaffected–like the VF setting in the experimental experiment–the blood pressure was dynamic and a significant difference between the arterial and venous blood pressure was observed (independent of their volume status, Figure 3b). Without reflex mechanisms, the normalized RMSE between simulations and experimental data was ~15%. Incorporation of reflex mechanisms reduced this error to <5%, substantially improving fidelity and confirming their importance for accurately reproducing experimental hemodynamic responses.

## 4 DISCUSSION

Our study demonstrates that blood pressure fluctuations following VF‐induced cardiac arrest challenge the assumed stability of MCFP. Unlike the stable equilibration of arterial and venous pressures observed after barbiturate overdose, VF resulted in a sustained retrograde pressure gradient between arterial and venous compartments. These differences emphasize the critical impact of the method of cardiac arrest induction on circulatory dynamics.

Furthermore, in silico simulations revealed that vascular reflex mechanisms play a key role in explaining these hemodynamic patterns. Agreement with experimental data was limited in the absence of reflex mechanisms (normalized RMSE ~15%), whereas their inclusion improved performance to <5%, demonstrating substantially enhanced model fidelity. This strongly suggests that zero‐flow measurements, influenced by reflex activity and the method of cardiac arrest, do not necessarily represent a universal or stable circulatory pressure, as postulated for MCFP.

### 4.1 Blood pressure after cardiac arrest

The persistence of ongoing blood flow after VF onset, previously demonstrated at the microcirculatory level (Fries 2006), suggests that dynamic blood pressure changes may arise from the presence of vascular reflex mechanisms and the contributions of pressure‐dependent, shear stress‐dependent, and metabolic vasoactive responses, particularly ischemia (Carlson et al., 2008). Our in silico simulations confirmed this notion by reproducing the dynamic blood pressure curves observed experimentally when simulated reflex mechanisms were activated. This highlights the critical role of vascular reflex activity in modulating post‐arrest hemodynamics.

Ischemia‐induced changes in vessel wall properties, such as disruption of the actin cytoskeleton in smooth muscle, also contribute to these dynamics by altering vascular elastance and tone (Baldassarre et al., 2001; Cipolla et al., 2001). These changes impact vascular compliance differentially in arterial and venous compartments, compounding the effects of intravascular volume shifts on blood pressure. Vasoactive agents such as noradrenaline can further modulate these dynamics by increasing venous tone and shifting blood from the unstressed to the stressed compartment (Åneman et al., 2021). The direct vascular effects of barbiturates, in contrast, appear to mitigate these ischemia‐driven changes, resulting in more stable pressure profiles. The in silico model did not account for anesthetic effects, which may influence baseline hemodynamics but would not eliminate the contribution of vascular reflex mechanisms.

In addition to dynamic pressure changes, we observed a persistent retrograde pressure difference after cardiac arrest, which suggests the presence of high resistance or a functional blockage between arterial and venous circulations. This is supported by the ability of venous valves to withstand retrograde pressures and the potential influence of a Starling resistor mechanism at the arteriolar level (Carlson et al., 1959; Magder, 1990). Such mechanisms may explain the observed positive perfusion pressure at zero flow, even in the absence of active circulation. However, the lack of intra‐abdominal pressure measurements limits our ability to fully assess the physiological range of retrograde pressures in piglets (Diaz, 2015). It should also be noted that vasoactive agents, such as noradrenaline, can directly increase mean circulatory filling pressure by enhancing venous tone and shifting blood from the unstressed to the stressed volume compartment, underlining the sensitivity of these measurements to pharmacological influences.

### 4.2 Methodological differences in MCFP measurement

The challenges of measuring MCFP are underscored by variability in experimental setups, including differences in the method of cardiac arrest (e.g., VF, acetylcholine, or barbiturate overdose), timing of blood pressure measurements, and the use of artificial shunts between arterial and venous vessels. These methodological differences significantly influence zero‐flow pressure measurements and complicate cross‐study comparisons (Aneman et al., 2024; Persichini et al., 2022).

Historically, Guyton's seminal work utilized an arterial‐to‐venous shunt to rapidly equilibrate pressures after VF‐induced cardiac arrest (Guyton et al., 1954). While effective in creating a stable MCFP measurement, this artificial equilibration deviates from natural conditions, where reflex activity and vascular compliance influence hemodynamics. Our study confirms that such natural conditions result in non‐equilibrated pressures, challenging the notion of a stable MCFP, especially without shunt‐assisted equilibration.

Moreover, our findings align with studies by Gaddis and colleagues, who demonstrated that the method of cardiac arrest induction directly influences observed pressures (Gaddis et al., 1986). Similarly, Repessé et al. observed significant dispersion in intravascular pressures after death, influenced by post‐mortem alterations in vasomotor tone (Repessé et al., 2017). These findings collectively highlight the critical role of experimental design in MCFP measurements and underscore the need for caution when interpreting results across studies.

### 4.3 Implications

Applying MCFP measurements from post‐arrest conditions to a beating‐heart scenario is inherently problematic. Measuring venous return pressure post‐arrest is akin to estimating fuel consumption in a stalled engine—the underlying dynamics do not match normal circulation.

Despite these limitations, zero‐flow measurements have historically been valuable for understanding venous return and stressed volume, and they remain conceptually important as the foundation of Guyton's model. Clinically, surrogate measures of MCFP have been explored to assess intravascular volume status and fluid responsiveness, but their applicability is constrained by the impossibility of achieving true zero‐flow in patients (Cooke et al., 2018; Maas 2012).

Our findings challenge the assumption that MCFP provides meaningful clinical information in dynamic conditions. The nonlinear pressure‐volume properties of the vasculature, combined with the structural complexity of the circulatory system, raise concerns about the validity of MCFP in beating‐heart physiology (Laou et al., 2023). If external pressure, blood volume, and vascular elasticity all influence MCFP, it becomes unclear whether its measurement under zero‐flow conditions has true clinical relevance.

Beyond this conceptual limitation, our study confirms the transformative potential of computational modeling (Quinn et al., 2023). By accurately reproducing in vivo hemodynamic responses, in silico simulations provide a mechanistic understanding of circulatory dynamics and could ultimately reduce reliance on large animal models in translational research (Mamorita et al., 2024). These computational models allow for controlled, reproducible studies, supporting more ethical and sustainable approaches to cardiovascular research while maintaining, or even enhancing, scientific rigor. When combined with patient‐specific data, such models can form the basis of digital twin approaches, integrating multiple data sources for individualized assessment of a patient's volume status (Venkatesh et al., 2022; Warnaar et al., 2023).

### 4.4 Limitations

Our study was not designed to replicate Guyton's original experiment, but rather to examine methodological limitations of MCFP measurement. The absence of a pressure difference in Guyton's shunt‐based studies can likely be attributed to artificial equilibration methods, which were not present in our experiments.

Although we took extensive precautions in zeroing, leveling, and calibrating the pressure systems, measurement errors can never be fully excluded. Additionally, our study focused on short‐term hemodynamic responses after cardiac arrest. Longer‐term effects on vascular tone and compliance were not assessed, and observations were therefore limited to 10 minutes, within which pressures reached a plateau. The small sample size, particularly in the hypovolemic VF group, is another limitation.

From a modeling perspective, in silico simulations, while powerful, simplify biological complexity (Fresiello & Donker, 2024). Although our model closely matched experimental data when reflex mechanisms were included (normalized RMSE <5%), it remains a deterministic system, constrained by predefined parameters. It should also be noted that CVSim was developed to capture human physiology, while the experimental data in this study were obtained from animals. Future studies should integrate patient‐specific data to further refine these models for clinical application (Bruynseels et al. 2018).

## 5 CONCLUSION

Our findings demonstrate that the method used to determine MCFP significantly alters recorded pressures, casting doubt on its clinical relevance as a stable measure of filling status. Reflex mechanisms, ischemia, and cardiac arrest induction method all influence MCFP measurements, suggesting that alternative approaches may be needed to assess circulatory function in critically ill patients.

Importantly, in silico simulations provided valuable mechanistic insights and closely replicated experimental findings when reflex mechanisms were included. These models offer a powerful tool for studying circulatory dynamics while promoting ethical, cost‐effective research by reducing reliance on large animal models. Moving forward, computational approaches should be further developed to complement experimental and clinical studies, ultimately improving our understanding of hemodynamic physiology.

## FUNDING INFORMATION

This work was supported by institutional funding and by ZonMw “More knowledge with less animals”.

## CONFLICT OF INTEREST STATEMENT

None declared.

## ETHICS STATEMENT

This experiment was performed after approval by the local ethics committee on animal research of the Radboud University Nijmegen Medical Center (RUNMC License number RU‐DEC 2014‐246) and in full compliance with Dutch and European legal requirements on the use and protection of laboratory animals.

## CONSENT

Not applicable.

## Data Availability

The materials described in this manuscript, including all relevant raw data, is freely available to any scientist wishing to use them for non‐commercial purposes.

## References

[phy270599-bib-0001] Aneman, A. , Skrifvars, M. B. , & Ameloot, K. (2024). Venous return physiology applied to post‐cardiac arrest haemodynamic management: A post hoc analysis of the NEUROPROTECT trial. Intensive Care Medicine Experimental, 12(1), 70. 39138823 10.1186/s40635-024-00657-0 PMC11322455

[phy270599-bib-0002] Åneman, A. , Wilander, P. , Zoerner, F. , Lipcsey, M. , & Chew, M. S. (2021). Vasopressor responsiveness beyond arterial pressure: A conceptual systematic review using venous return physiology. Shock, 56(3), 352–359. 33756500 10.1097/SHK.0000000000001762

[phy270599-bib-0003] Baldassarre, D. , Amato, M. , Palombo, C. , Morizzo, C. , Pustina, L. , & Sirtori, C. R. (2001). Time course of forearm arterial compliance changes during reactive hyperemia. American Journal of Physiology. Heart and Circulatory Physiology, 281(3), H1093–H1103. 11514275 10.1152/ajpheart.2001.281.3.H1093

[phy270599-bib-0004] Bruynseels, K. , Santoni de Sio, F. , & van den Hoven, J. (2018). Digital twins in health care: Ethical implications of an emerging engineering paradigm. Frontiers Media SA, 9, 31. 10.3389/fgene.2018.00031 PMC5816748 29487613

[phy270599-bib-0005] Carlson, B. E. , Arciero, J. C. , & Secomb, T. W. (2008). Theoretical model of blood flow autoregulation: Roles of myogenic, shear‐dependent, and metabolic responses. American Journal of Physiology. Heart and Circulatory Physiology, 295(4), H1572–H1579. 18723769 10.1152/ajpheart.00262.2008 PMC2593503

[phy270599-bib-0006] Carlson, L. B. H. , Stacey, B. D. , & Curtis, H. (1959). Tissue pressure and critical closing pressure in the dog kidney. American Journal of Physiology, 196(5), 1132–1134. 13649946 10.1152/ajplegacy.1959.196.5.1132

[phy270599-bib-0007] Cipolla, M. J. , Lessov, N. , Hammer, E. S. , & Curry, A. B. (2001). Threshold duration of ischemia for myogenic tone in middle cerebral arteries: Effect on vascular smooth muscle actin. Stroke, 32(7), 1658–1664. 11441216 10.1161/01.str.32.7.1658

[phy270599-bib-0008] Cooke, K. , Sharvill, R. , Sondergaard, S. , & Aneman, A. (2018). Volume responsiveness assessed by passive leg raising and a fluid challenge: A critical review focused on mean systemic filling pressure. Anaesthesia, 73(3), 313–322. 29171669 10.1111/anae.14162

[phy270599-bib-0009] deBoer, R. W. , Karemaker, J. M. , & Strackee, J. (1987). Hemodynamic fluctuations and baroreflex sensitivity in humans: A beat‐to‐beat model. American Journal of Physiology. Heart and Circulatory Physiology, 3(8), H680–H689. 10.1152/ajpheart.1987.253.3.H680 3631301

[phy270599-bib-0010] Diaz, F. (2015). Influence of tidal volume on pulse pressure variation and stroke volume variation during experimental intra‐abdominal hypertension. BMC Anesthesiology, 15, 127. 26395001 10.1186/s12871-015-0105-x PMC4579832

[phy270599-bib-0011] Fresiello, L. , & Donker, D. W. (2024). All models are wrong but some provide seemingly surprising mechanistic insights into the complexity of venoarterial extracorporeal membrane oxygenation. Perfusion, 40, 2676591241263908. 10.1177/02676591241263908 39259606

[phy270599-bib-0012] Fries, M. (2006). Microcirculation during cardiac arrest and resuscitation. Critical Care Medicine, 34(12 Suppl), S454–S457. 17114977 10.1097/01.CCM.0000247717.81480.B2

[phy270599-bib-0013] Gaddis, M. L. , Rothe, C. F. , Tunin, R. S. , Moran, M. , & MacAnespie, C. (1986). Mean circulatory filling pressure: Potential problems with measurement. The American Journal of Physiology, 251(4 Pt 2), H857–H862. 3766762 10.1152/ajpheart.1986.251.4.H857

[phy270599-bib-0014] Guyton, A. C. , Jones, C. E. , & Coleman, T. G. (1973). Circulatoy physiology: Cardiac output and its regulation (2nd ed.). Saunders.

[phy270599-bib-0016] Guyton, A. C. , Lindsey, A. W. , & Kaufmann, B. N. (1955). Effect of mean circulatory filling pressure and other peripheral circulatory factors on cardiac output. American Journal of Physiology‐Legacy Content, 180(3), 463–468. 10.1152/ajplegacy.1955.180.3.463 14376522

[phy270599-bib-0015] Guyton, A. C. , Polizo, D. , & Armstrong, G. G. (1954). Mean circulatory filling pressure measured immediately after cessation of heart pumping. The American Journal of Physiology, 179(2), 261–267. 13218155 10.1152/ajplegacy.1954.179.2.261

[phy270599-bib-0017] Heldt, T. (2010). CVSim: An open‐source cardiovascular simulator for teaching and research. Open Pacing, Electrophysiology & Therapy Journal, 3, 45–54. PMC3178445 21949555

[phy270599-bib-0018] Laou, E. , Papagiannakis, N. , Sarchosi, S. , Kleisiaris, K. , Apostolopoulou, A. , Syngelou, V. , Kakagianni, M. , Christopoulos, A. , Ntalarizou, N. , & Chalkias, A. (2023). The use of mean circulatory filling pressure analogue for monitoring hemodynamic coherence: A post‐hoc analysis of the SPARSE data and proof‐of‐concept study. Clinical Hemorheology and Microcirculation, 84(1), 19–32. 36846992 10.3233/CH-221563

[phy270599-bib-0019] Maas, J. J. (2012). Estimation of mean systemic filling pressure in postoperative cardiac surgery patients with three methods. Intensive Care Medicine, 38, 1452–1460. 22584797 10.1007/s00134-012-2586-0 PMC3423572

[phy270599-bib-0020] Magder, S. (1990). Starling resistor versus compliance. Which explains the zero‐flow pressure of a dynamic arterial pressure‐flow relation? Circulation Research, 67(1), 209–220. 2364491 10.1161/01.res.67.1.209

[phy270599-bib-0021] Mamorita, N. , Takeuchi, A. , & Kamata, H. (2024). An interactive simulator to deepen the understanding of Guyton's venous return curve. The Journal of Physiological Sciences, 74(1), 21. 38555424 10.1186/s12576-024-00912-9 PMC10981291

[phy270599-bib-0022] Moody, G. B. , Mark, R. G. , & Goldberger, A. L. (2000). PhysioNet: A research resource for studies of complex physiologic and biomedical signals. Computers in Cardiology, 27, 179–182. 14632011

[phy270599-bib-0023] Persichini, R. , Lai, C. , Teboul, J.‐L. , Adda, I. , Guérin, L. , & Monnet, X. (2022). Venous return and mean systemic filling pressure: Physiology and clinical applications. Critical Care, 26(1), 150. 35610620 10.1186/s13054-022-04024-x PMC9128096

[phy270599-bib-0024] Quinn, A. R. J. , Saxby, D. J. , Yang, F. , de Sousa, A. C. C. , & Pizzolato, C. (2023). A digital twin framework for robust control of robotic‐biological systems. Journal of Biomechanics, 152, 111557. 37019066 10.1016/j.jbiomech.2023.111557

[phy270599-bib-0025] Repessé, X. , Charron, C. , Geri, G. , Aubry, A. , Paternot, A. , Maizel, J. , Slama, M. , & Vieillard‐Baron, A. (2017). Impact of positive pressure ventilation on mean systemic filling pressure in critically ill patients after death. Journal of Applied Physiology (1985), 122, 1373–1378. 10.1152/japplphysiol.00958.2016 28360123

[phy270599-bib-0026] Steptoe, A. , Rüddel, H. , & Neus, H. (1985). Clinical and methodological issues in cardiovascular psychophysiology. Springer Berlin Heidelberg.

[phy270599-bib-0027] van Loon, L. M. , van der Hoeven, H. , Veltink, P. H. , & Lemson, J. (2020). The inspiration hold maneuver is a reliable method to assess mean systemic filling pressure but its clinical value remains unclear. Annals of Translational Medicine, 8(21), 1390. 33313135 10.21037/atm-20-3540 PMC7723632

[phy270599-bib-0028] Venkatesh, K. P. , Raza, M. M. , & Kvedar, J. C. (2022). Health digital twins as tools for precision medicine: Considerations for computation, implementation, and regulation. NPJ Digital Medicine, 5(1), 150. 36138125 10.1038/s41746-022-00694-7 PMC9500019

[phy270599-bib-0029] Warnaar, R. S. P. , Mulder, M. P. , Fresiello, L. , Cornet, A. D. , Heunks, L. M. A. , Donker, D. W. , & Oppersma, E. (2023). Computational physiological models for individualised mechanical ventilation: A systematic literature review focussing on quality, availability, and clinical readiness. Critical Care, 27(1), 268. 37415253 10.1186/s13054-023-04549-9 PMC10327331

[phy270599-bib-0030] Zong, W. (2003). An open‐source algorithm to detect onset of arterial blood pressure pulses. Computers in Cardiology, 1, 259–262.

